# Memory accessibility as a cue for perceived importance

**DOI:** 10.3758/s13421-025-01772-3

**Published:** 2025-08-06

**Authors:** Dillon H. Murphy, Aikaterini Stefanidi, Gene A. Brewer

**Affiliations:** 1 Arizona State University, Tempe, AZ USA; 2 Department of Psychology, University of California, Riverside, CA USA

**Keywords:** Judgments of importance, Retrieval, Accessibility, Forgetting, Feeling of knowing

## Abstract

People frequently rely on subjective assessments of importance to navigate daily decisions, yet the psychological underpinnings of these judgments are not fully understood. Crucially, non-diagnostic factors, such as memory accessibility, may skew these evaluations. The present study examined the interplay between memory outcomes and judgments of importance. Participants engaged in a memory test involving 20 scientific theories, followed by assessments of each theory’s importance. Results revealed a bias whereby successfully recalled theories were deemed more important than those not recalled. Additionally, even in the case of retrieval failure, metacognitive feelings of knowing positively correlated with importance judgments. Finally, when memory was tested via recognition, which lowers retrieval difficulty, this importance bias was diminished, indicating that the effort or challenge of retrieval may be used as a cue for importance. Across these experiments, a consistent pattern emerged (recalled information was considered more important than forgotten information) that aligns with the hypothesis that memory accessibility and subjective judgments of importance are intertwined. Thus, people may deem things they remember as having higher importance and things they forget as having less importance, based in part on the degree of memory accessibility which is not necessarily a valid indicator of the true status of that information’s value.

## Introduction

Every day, people make judgments about what is important, yet the scientific understanding of how these judgments are formed remains incomplete. Individuals often prioritize items to purchase and create “to-do” lists, allocating their time based on perceived importance and urgency. Similarly, judgments of importance play a crucial role in the realm of science. Notably, the US Department of Health and Human Services (2013) explicitly requests peer reviewers to assess the importance of proposed or published research. However, these judgments are sometimes influenced by non-diagnostic factors such as the country of origin or the institution where the research was conducted. The current study examined how long-term memory processes play a critical role in evaluating the importance of information.

Prior research has used the value-directed remembering paradigm (e.g., Castel et al., 2002; Elliott et al., 2020; Murphy et al., 2021) to examine how the value of information influences memory performance. In a typical value-directed remembering task, participants are given a list of words to study for a later memory test with each word assigned a specific “value” represented by a number next to the word (e.g., chicken 4). Participants are instructed that on a subsequent memory test, they will be awarded the point value associated with a word if they can remember it. The participant’s goal is to maximize their accumulated points via recall (or however memory is assessed). Therefore, some words are more important to remember than others (i.e., learners should prioritize the encoding and retrieval of high-value items). Prior work using these value-directed remembering paradigms has demonstrated that participants typically recall more high-value words than low-value words (for a review, see Knowlton & Castel, 2022).

While point values can drive encoding processes, value-based remembering also involves retrieval processes. For example, prior work has demonstrated that participants tend to initiate recall with the highest-valued words and organize retrieval in a manner that prioritizes valuable information, with high-value items being recalled before low-value items (Murphy & Castel, 2021c; Stefanidi et al., 2018). Additionally, participants maximize memory utility by efficiently recalling high-value words and grouping them together during recall transitions (Murphy et al., 2022). This suggests that value influences the organization of memory retrieval, but it is also possible that these effects reflect encoding-based processes, such as deeper or more elaborate encoding of high-value items, which could lead to their prioritized retrieval. Thus, while the observed output patterns imply strategic retrieval, they may also be driven by encoding-related mechanisms.

Similar to objective point values guiding memory, prior work indicates that information of greater subjective importance is more likely to be remembered than less important information (see Murphy & Castel, 2020, 2021a, b, 2022a, b, 2023; Murphy et al., 2023a, b). For example, Murphy et al. (2023b) presented participants with words along a theme (e.g., going on a camping trip) and had participants rank the items according to importance. Results revealed that participants better remembered the items they considered important relative to the items they considered less important. This suggests that an adaptive and effective memory system operates by selectively encoding and successfully retaining important information while disregarding and forgetting non-important information.

Evidence suggests that we are more likely to retain and recall information that holds value or significance to us whether it is personally relevant, emotionally impactful, or crucial to our goals and needs. When we encounter situations where we need to recall this vital information and it is successfully retrieved, the perceived relationship between importance and memory is reinforced, creating a kind of feedback loop that strengthens the link between the perceived importance of information and our memory’s effectiveness. Consequently, we propose that individuals naturally develop a robust and intricate association between the perception of importance and memory performance. Specifically, we suggest an *importance bias* whereby the accessibility of information in memory predicts our perception of that information’s importance.

The availability heuristic refers to the tendency to rely on examples that come easily to mind when estimating the frequency or probability of events (Schwarz et al., 1991; Tversky & Kahneman, 1973). Although this heuristic typically influences judgments about how common or likely something is, a similar process may underlie evaluations of importance. Specifically, if something is easily remembered, people may infer not only that it is more common, but also that it is more meaningful. For example, after hearing about airplane accidents on the news, people may overestimate the risk of flying and also assign greater importance to the event, simply because such instances are more accessible in memory than the countless uneventful flights. In this way, memory accessibility may bias both probability and importance judgments.

Prior work has provided initial evidence that judgments of value or importance can be shaped by memory performance, but the underlying mechanisms differ. For example, Castel et al. (2012) used a value-directed remembering paradigm and found that participants were more likely to associate higher point values with successfully recalled words and lower values with forgotten words – a pattern they termed the “forgetting bias.” This reflects a negative or absence-based heuristic, where the lack of memory leads people to infer lower importance. In contrast, Filiz and Dobbins (2024) demonstrated a positive or presence-based heuristic, showing that recognition strength, such as familiarity or fluency, served as a cue for inferring higher value. This finding aligns with the recognition heuristic (Goldstein & Gigerenzer, 2002), suggesting that accessible information is perceived as more valuable. Together, these studies support the idea that memory accessibility influences perceived importance, either through devaluation of forgotten items or inflation of remembered ones.

Although Castel et al. (2012) uncovered an intriguing metacognitive bias and Filiz and Dobbins (2024) demonstrated a recognition heuristic, participants were not explicitly prompted to make subjective judgments of importance regarding the information they successfully or unsuccessfully remembered. Instead, the focus of the study was on having participants recall the objective point value associated with each word during the study phase. Incorporating direct assessments of participants’ subjective judgments of importance may offer a more holistic understanding of the complex interplay between memory, metacognition (our ability to monitor and regulate our cognitive processes, including memory and decision-making; Dunlosky et al., 2016; Nelson, 1996; Rhodes, 2016), and the perception of importance.

Again, Castel et al. (2012) demonstrated that individuals tend to mistakenly believe that forgotten information is of lesser value. However, the success or failure of retrieving an item does not necessarily reflect or modify the item’s subjective importance. Thus, we propose the existence of a metacognitive bias such that people tend to remember information with higher inherent value or importance, but in the absence of successful remembering, people may incorrectly conclude that the information being searched for is of less value or importance. By delving into this potential importance bias, we can gain a deeper understanding of how memory processes influence the way we perceive the importance of information.

### The current study

We theorized that the perceived importance of remembered information, compared to forgotten information, may be predicted by the accessibility of information in memory. Specifically, we hypothesized that when information is more readily accessible in memory, it tends to be considered more important, even if it cannot be retrieved at a given moment. Feelings-of-knowing judgments, as a metacognitive measure, provide valuable insights into memory accessibility by reflecting an individual’s awareness of their memory capabilities and their confidence in successfully recalling specific information (Hart, 1965; Hertzog et al., 2010; Irak et al., 2019; Koriat, 1993; Schacter, 1983). Thus, feelings of knowing serve as cues to the accessibility of information in memory, even before an actual retrieval attempt is made (Koriat, 1995, 2005). If retrieval processes bias subjective evaluations of importance, then the more accessible information is in memory, the more important it should be perceived to be. In the current study, we aim to provide evidence of this importance bias such that the accessibility of information in memory predicts the perceived importance of the given information.

To investigate potential biases in subjective judgments of importance as a function of the success or failure of memory retrieval, we asked participants to study several scientific theories (e.g., Socioemotional Selectivity Theory: As people age and their perceived time left in life decreases, people become increasingly selective, investing greater resources in emotionally meaningful goals and activities. This motivational shift also influences cognitive processing by shifting focus from information-seeking goals to emotional goals) which they would later be tested on (given the definition, participants were asked to recall the name of the theory). Following the test for each theory, participants rated the importance of the theory for our understanding of the world on a continuous scale from 0 (not at all important) to 100 (very important). We expected participants to rate correctly remembered theories as more important than forgotten theories.

## Experiment 1

In Experiment 1, we investigated the relationship between memory retrieval and subjective judgments of importance. During the study phase, participants were asked to learn and study the definitions of 20 scientific theories. In the test phase, participants were presented with the definitions of the scientific theories and were instructed to recall the name of each theory. After responding to each item on the memory test, participants were asked to provide subjective judgments of importance for each of the scientific theories. Specifically, participants rated the importance of each theory on a scale from 0 to 100. Lastly, participants were shown all 20 theories again and were asked to rate how familiar they were with each theory before the study to control for prior exposure and domain knowledge of the theories. Again, we expected participants to illustrate a bias in their subjective importance ratings such that successfully recalled theories would be deemed more important than theories where participants experience a retrieval failure.

### Method

#### Participants

In each experiment, participants were recruited from the Arizona State University (ASU) Human Subjects Pool, were tested online, received course credit for their participation, and were excluded from analysis if they admitted to cheating (e.g., writing down answers) in a post-task questionnaire (they were told they would still receive credit if they cheated). Participants were also excluded if they did not attest to giving their best effort on the task. In each experiment, we aimed to collect around 100 participants per condition. The sample size was selected based on prior exploratory research and the expectation of detecting a medium effect size. In Experiment 1, after exclusions, participants were 103 undergraduate students (*M*_*age*_ = 19.07, *SD*_*age*_ = 2.01 years) – three participants were excluded for admitting to cheating, and no participants were excluded for not attesting to give their best effort on the task.

### Materials

Twentyscientific theories were chosen from various scientific fields (psychology, chemistry, physics, economics, etc.; all stimuli are available on the Open Science Framework, see *Data availability*). The selected theories were chosen because they were expected to be novel to participants. Each theory’s definition was summarized in a few sentences. Participants saw the definition on the left side of the screen paired with the theory name on the right side of the screen.

### Procedure

At the beginning of the task, participants were asked to

1. Be prepared to complete the study in a single, 30-min session (i.e., no prolonged breaks),
2. Complete this study in an environment with minimal distraction (i.e., put away extra devices, close unneeded tabs, and turn off any music or TV in the background),
3. Complete the study on a laptop or desktop computer (i.e., do NOT use a mobile phone or a tablet), and
4. If in the presence of other people, try to move somewhere more secluded.

Participants were then asked, “Please give your full effort on this task! PLEASE do not use any external aides and thus use ONLY YOUR OWN memory to complete the study.” Then, participants were told “This task will be very difficult. We do not expect you to remember everything; you will likely only be able to remember some of the information. Please do not cheat by taking pictures of the screen, writing things down, or using any other aid to complete this task.” Finally, we again asked participants that they do not cheat on this task by using any memory aids and that they give their full effort to this task. They then had to attest that they would not cheat and that they would give their best effort.

Participants were instructed that they would be studying a series of scientific theories for a later memory test and were asked to study them as if they were studying for a typical course examination. Each theory was presented one at a time, and the order of the theories was randomized for each participant. Study time was self-paced, allowing participants to study each theory as long as necessary. Immediately after the study phase, participants completed a 1-min distraction task requiring them to rearrange the digits of several three-digit numbers in descending order (e.g., 123 would be rearranged to 321). Participants were given 3 s to view each of the three-digit numbers and subsequently rearrange the digits.

For the cued recall test, participants were given each theory’s definition and were asked to retrieve the name of the theory. Participants were not given feedback or the correct answer following their responses. The order of the theories on the test was randomized, and the test phase was self-paced. After each retrieval attempt, participants were asked to rate the importance of that theory. Specifically, they were asked to rate “How important is this theory for our understanding of the world?” Participants made their rating on a scale ranging from 0 (not important) to 100 (very important). Judgments of importance were self-paced.

At the end of the memory test, participants were told that they would be presented with each theory and definition again. After viewing each one, participants rated their familiarity with each theory from before they had participated in the study. Specifically, participants rated their prior familiarity with each theory on a scale from 0 (not familiar at all) to 100 (very familiar). The order of the theories was again randomized, and familiarity ratings were self-paced.

### Results

To examine importance judgments for each theory as a function of whether it was correctly recalled, we parameterized a multilevel model (MLM) to account for the hierarchical structure of the data, with items (theories) nested within individual participants. Participants may have varied in their prior familiarity with the scientific theories, which could influence both recall accuracy and importance judgments. To control for these individual differences in prior knowledge, we included participants’ familiarity ratings as a covariate in our models. In addition, we included random intercepts for both participants and items (i.e., the individual theories) to account for potential variability in memorability and perceived importance across stimuli (Judd et al., 2012). This modeling approach allows us to isolate the effects of retrieval success and metacognitive variables on importance judgments while controlling for stimulus-specific and participant-specific variability.

**Fig. 1:**
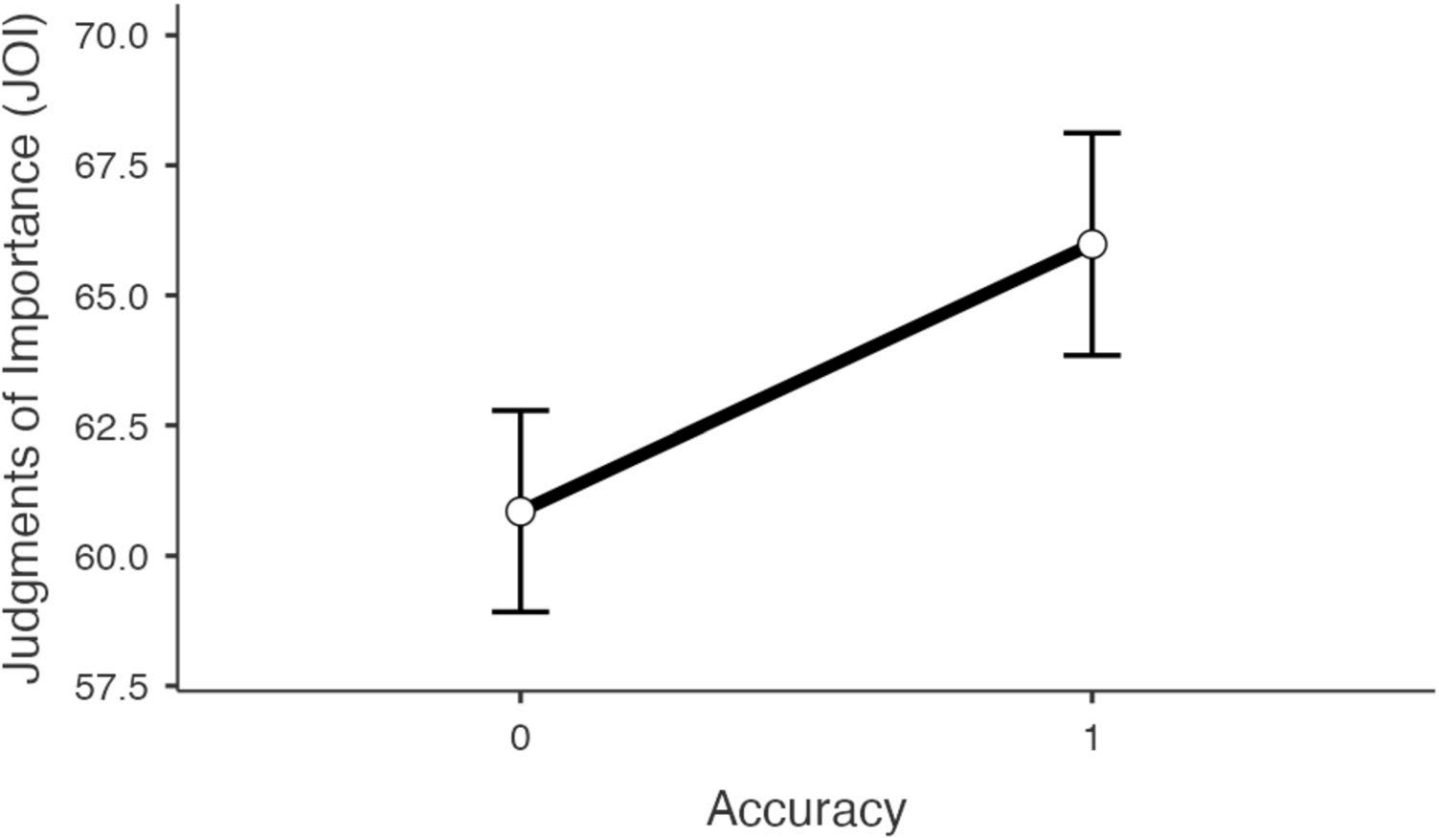
Judgments of importance (JOIs) as a function of recall accuracy while controlling for each participant’s prior familiarity with each theory in Experiment 1. Error bars reflect the standard error

Judgments of importance (JOIs) as a function of correctness while controlling for familiarity ratings are shown in Fig. 1. A mixed MLM with item-level JOIs modeled as a function of recall accuracy and familiarity revealed that recall accuracy significantly predicted JOIs (*t*(2009) = 4.25, *p* <.001, Estimate: 5.13), such that correctly recalled theories were rated as more important (*M* = 67.14, *SD* = 16.80) than theories that were not correctly recalled (*M* = 60.93, *SD* = 17.94). Familiarity also significantly predicted JOIs (*t*(2046) = 4.71, *p* <.001, Estimate:.08), such that theories that participants were more familiar with were rated as more important than theories that participants were less familiar with. Accuracy did not interact with familiarity (*t*(1985) = -.10, *p* =.918, Estimate: -.00).

We also examined whether there were potential item effects (e.g., whether certain theories were both highly important and memorable or vice versa). However, the average accuracy for each theory did not correlate with the average JOI for each theory (*r* =.08, *p* =.735). This further suggests that the observed relationship between JOIs and accuracy is influenced by retrieval success.

### Discussion

In Experiment 1, the theories for which participants were able to correctly recall the names were rated as more important than the theories that they could not recall, even when controlling for prior familiarity. This suggests a notable relationship between memory accessibility and subjective evaluations of importance, aligning with our hypothesis and providing evidence for the proposed importance bias.

## Experiment 2

Participants in Experiment 1 regarded remembered information as more important than forgotten information. If retrieval success biases judgments of importance, then a particular theory should be judged as being less important before the memory test compared to after the memory test (or, more specifically, after recall success). As such, it is possible that perceived importance was driving encoding in Experiment 1 and participants’ importance ratings at recall reflected differential encoding according to perceived importance rather than illustrating a retrieval bias whereby remembered information is considered more important than forgotten information. As such, in Experiment 2, we examined whether the effects from Experiment 1 hold when participants consider each theory’s importance during encoding. Specifically, we directly compared participants making importance ratings at recall only with participants making importance ratings during encoding as well as at recall. We expected to replicate the effects of Experiment 1 even when controlling for importance judgments at encoding.

### Method

#### Participants

After exclusions, participants were 205 undergraduate students (*M*_*age*_ = 18.98, *SD*_*age*_ = 2.53 years) – two participants were excluded for cheating and none were excluded for not agreeing to give their full effort.

#### Materials and procedure

For participants making importance ratings during recall only (*n* = 102), the materials and procedure in Experiment 2 were identical to those of Experiment 1. However, some participants (*n* = 103) also gave importance ratings during encoding. Specifically, after studying each theory, these participants were asked to rate the importance of that theory (self-paced) using the same rating procedure from Experiment 1. Participants were randomly assigned to experimental groups.

### Results

**Fig. 2:**
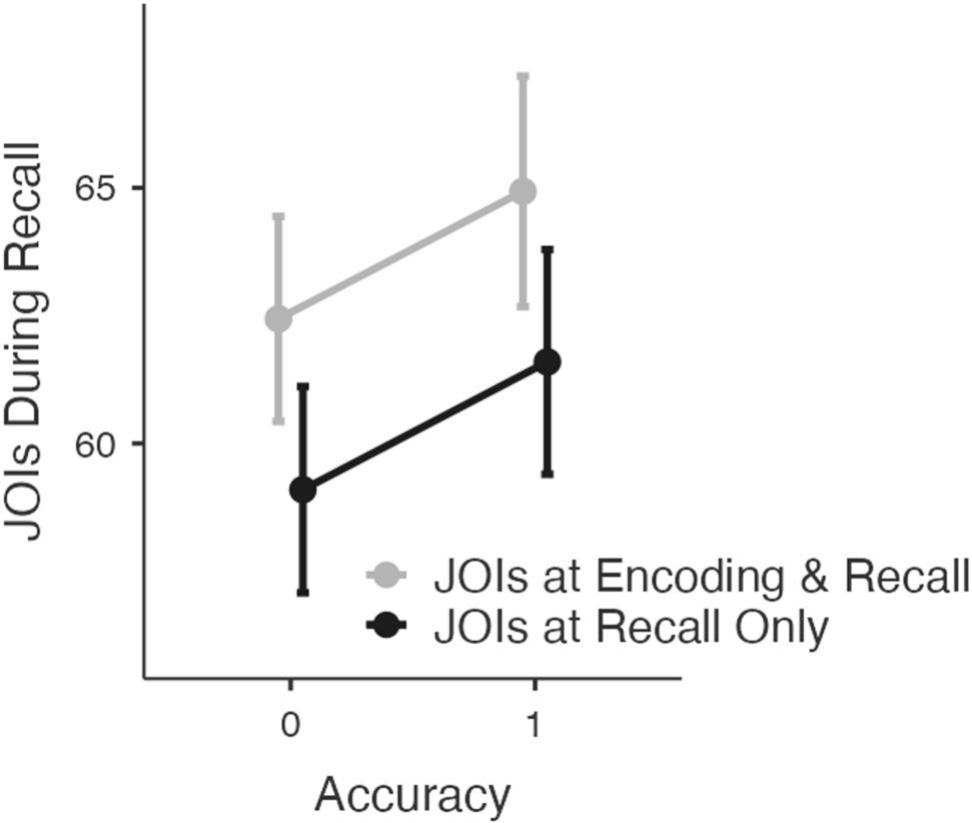
Judgments of importance (JOIs) from the recall phase as a function of accuracy while controlling for each participant’s prior familiarity with each theory in Experiment 2. Error bars reflect the standard error

Judgments of importance (JOIs) from the recall phase in each condition as a function of correctness while controlling for familiarity ratings are shown in Fig. 2. We began by conducting a mixed MLM with item-level JOIs from recall modeled as a function of condition (making JOIs at both encoding and recall or recall only), recall accuracy, and familiarity (since familiarity did not interact with accuracy in Experiment 1, for parsimony, we do not include interactions with familiarity in our subsequent models). Results revealed that memory accuracy significantly predicted JOIs at recall (*t*(4019) = 2.84, *p* =.005, Estimate: 2.50), such that theories that were correctly recalled were rated as more important (*M* = 65.60, *SD* = 18.83) than theories that were not correctly recalled (*M* = 60.36, *SD* = 19.40). Familiarity also significantly predicted JOIs at recall (*t*(4088) = 11.27, *p* <.001, Estimate:.13), such that the theories that participants were more familiar with were rated as more important during recall than the theories that participants were less familiar with. However, whether or not participants made JOIs at encoding did not predict JOIs at recall (*t*(220) = −1.32, *p* =.189, Estimate: −3.34) and condition did not interact with accuracy (*t*(3980) =.00, *p* =.997, Estimate:.01). Thus, we replicated the findings of Experiment 1 such that a theory was rated as more important following successful recall than if the name of the theory failed to be retrieved.

**Fig. 3:**
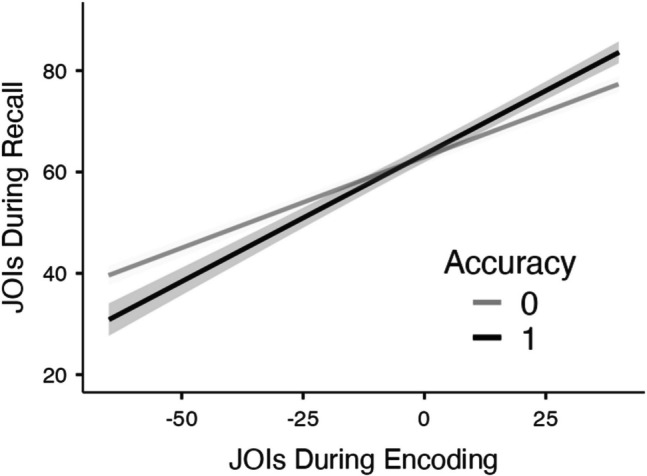
Judgments of importance (JOIs) from the recall phase as a function of JOIs at encoding for correct and incorrect retrieval attempts while controlling for each participant’s prior familiarity with each theory in Experiment 2. Standard errors are shown in gray. We note that due to model centering, the *x*-axis does not necessarily reflect the true scale of the variable

Next, we further examined participants who made JOIs at both encoding and recall. Specifically, in the previous model, we did not control for JOIs during the encoding phase. Figure 3 shows JOIs during recall as a function of JOIs at encoding for correct and incorrect retrieval attempts while controlling for familiarity. When JOIs at encoding are added (as well as the interaction between recall accuracy and JOIs at encoding), the effect of recall accuracy is no longer significant (*t*(2046) =.48, *p* =.631, Estimate:.56), such that remembered and forgotten theories were rated as similarly important. Additionally, JOIs at encoding predicted JOIs at recall (*t*(1992) = 18.22, *p* <.001, Estimate:.43), such that theories rated as more important during encoding were more likely to be rated as important during recall. However, JOIs at encoding interacted with recall accuracy (*t*(1965) = 3.35, *p* <.001, Estimate:.14). To explore this interaction, we analyzed the simple effects. Results revealed that JOIs during encoding were better predictors of JOIs at recall when the item was recalled (*t*(2003) = 12.47, *p* <.001, Estimate:.50) than when it was forgotten (*t*(1868) = 17.70, *p* <.001, Estimate:.36). This suggests that if participants recalled a theory correctly, their recall-phase judgment of importance tended to closely reflect their initial judgment at encoding (likely considering it important). However, for theories that were forgotten, the relationship between encoding- and recall-phase JOIs was weaker. This pattern suggests that successful memory retrieval strengthens the consistency between initial and later evaluations, perhaps because successful recall reinforces or validates the original (potentially high) importance judgment, but when participants fail to retrieve a theory, they may adjust their importance ratings at recall, perhaps downgrading its perceived importance.

As a follow-up analysis, we examined whether importance ratings from encoding changed at retrieval as a function of whether a theory was correctly recalled. Specifically, we conducted a mixed MLM with the difference between JOIs at encoding and JOIs at recall modeled as a function of recall accuracy. Results revealed that recall accuracy did not significantly predict the difference between JOIs at encoding and recall (*t*(1928) =.02, *p* =.984, Estimate:.03). Thus, for participants making JOIs at both encoding and recall, there was not a significant change in JOIs based on recall success.

We also examined whether JOIs at encoding show similar effects to JOIs at recall (i.e., higher JOIs for items that are remembered). Specifically, we conducted a mixed MLM with item-level JOIs from encoding modeled as a function of recall accuracy and familiarity. Results revealed that accuracy did not significantly predict JOIs at encoding (*t*(2031) = 1.07, *p* =.286, Estimate: 1.41). This indicates that subjective importance did not drive encoding (i.e., theories that were considered more important at encoding were not better recalled), which is reasonable given that participants had no incentive or instruction to selectively prioritize certain items during encoding. However, familiarity significantly predicted JOIs at encoding (*t*(2005) = 9.74, *p* <.001, Estimate:.16), such that theories that participants were more familiar with were rated as more important during encoding than theories participants were more familiar with. Overall, this analysis indicates that the importance bias observed at recall is not merely a reflection of pre-existing importance perceptions influencing encoding effort. Instead, this finding supports the idea that retrieval success may shape or bias importance judgments after the fact rather than simply reflecting earlier beliefs about what was important. This distinction is crucial in interpreting the directionality of the relationship between memory and perceived importance – it points toward retrieval processes playing a meaningful role in shaping importance judgments.

### Discussion

In Experiment 2, we examined whether the perceived importance of a theory at encoding could account for some of the effects observed in Experiment 1. While we did not find importance ratings at encoding to predict recall success, theories that participants successfully recalled were rated (after recall) as more important than those they did not, affirming the connection between memory retrieval and perceived importance. However, this effect dissipated when JOIs made at encoding were considered, and memory success/failure did not result in a significant change in importance ratings from encoding to recall, though participants’ initial importance ratings (JOIs at encoding) were more strongly aligned with their later ratings (JOIs at recall) when the theory was successfully remembered than when it was forgotten. This suggests that successful memory retrieval reinforces or validates prior importance judgments, whereas retrieval failure may prompt participants to downgrade the importance of the forgotten theory.

Overall, the results of Experiment 2 were mixed in terms of determining whether the observed importance bias is primarily driven by encoding or retrieval processes. Although we aimed to disentangle these factors by collecting importance judgments at encoding, the inherent interdependence of encoding and retrieval makes it difficult to isolate their respective contributions. For example, it could be the case that soliciting JOIs during encoding exerts its own bias on the recall process and subsequent importance ratings. Prior work has shown that soliciting metacognitive judgments such as JOIs during encoding can change what is remembered (e.g., Murphy & Castel, 2021a; Murphy et al., 2023a), a form of metacognitive reactivity (i.e., when making a judgment about something changes what or how much is remembered). Thus, to better understand the subjective importance of information retrieved from memory, we may need to further examine importance ratings with different levels of memory accessibility. To provide evidence that encoding is not determinative of this importance bias, Experiments 3 and 4 were designed to examine specific aspects of the retrieval process.

## Experiment 3

If the effects observed in Experiments 1 and 2 (i.e., an importance bias whereby remembered information is considered more important than forgotten information) are due to the accessibility of information in memory, then participants should show a similar bias even in the case of retrieval failure. During retrieval, feelings of knowing (a metacognitive judgment about the ability to retrieve specific elements from memory in the absence of redintegration, i.e., the completion of remembering) could also influence how we perceive importance. Specifically, even when participants experience retrieval failure, there may still be a form of an importance bias. As such, in the absence of redintegration, if importance judgments are positively related to feelings of knowing, this would provide further evidence that memory accessibility biases the perceived importance of information. As such, in Experiment 3, all participants only made importance ratings during retrieval (akin to Experiment 1) but also made a feeling-of-knowing judgment before providing their importance rating for each item during the test phase. Even on incorrect trials, we expected feeling-of-knowing judgments to positively relate to importance ratings such that the greater a given item’s accessibility in memory, the more important it is perceived to be.

### Method

#### Participants

Participants were 99 undergraduate students (*M*_*age*_ = 19.18, *SD*_*age*_ = 3.27 years) – no participants were excluded for cheating and none were excluded for not agreeing to give their full effort.

#### Materials and procedure

The encoding phase in Experiment 3 was identical to Experiment 1. After each retrieval attempt, participants made feeling of knowing judgments before making each importance rating (the importance rating procedure was the same as in Experiment 1). Specifically, after each retrieval attempt (but before making importance ratings), participants were asked: “Do you know the answer to the extent that you could pick the correct answer from among several choices in the future?” Participants were asked to respond on a scale from 0 (definitely will not be able to pick the correct answer) to 100 (definitely will be able to pick the correct answer). Feeling of knowing judgments were self-paced. Again, after the test phase, participants provided prior familiarity ratings for each theory.

### Results

We began by conducting a mixed MLM with item-level feeling-of-knowing judgments from recall modeled as a function of accuracy and familiarity. Results revealed that accuracy significantly predicted feelings of knowing (*t*(1950) = 19.30, *p* <.001, Estimate: 32.65), indicating that participants’ feeling-of-knowing judgments were accurate – participants gave higher feelings of knowing when the item was recalled than when it was not recalled. Familiarity also significantly predicted feelings of knowing (*t*(1895) = 8.44, *p* <.001, Estimate:.19), such that the theories that participants were more familiar with were rated as being closer to recalled than theories participants were less familiar with.

**Fig. 4:**
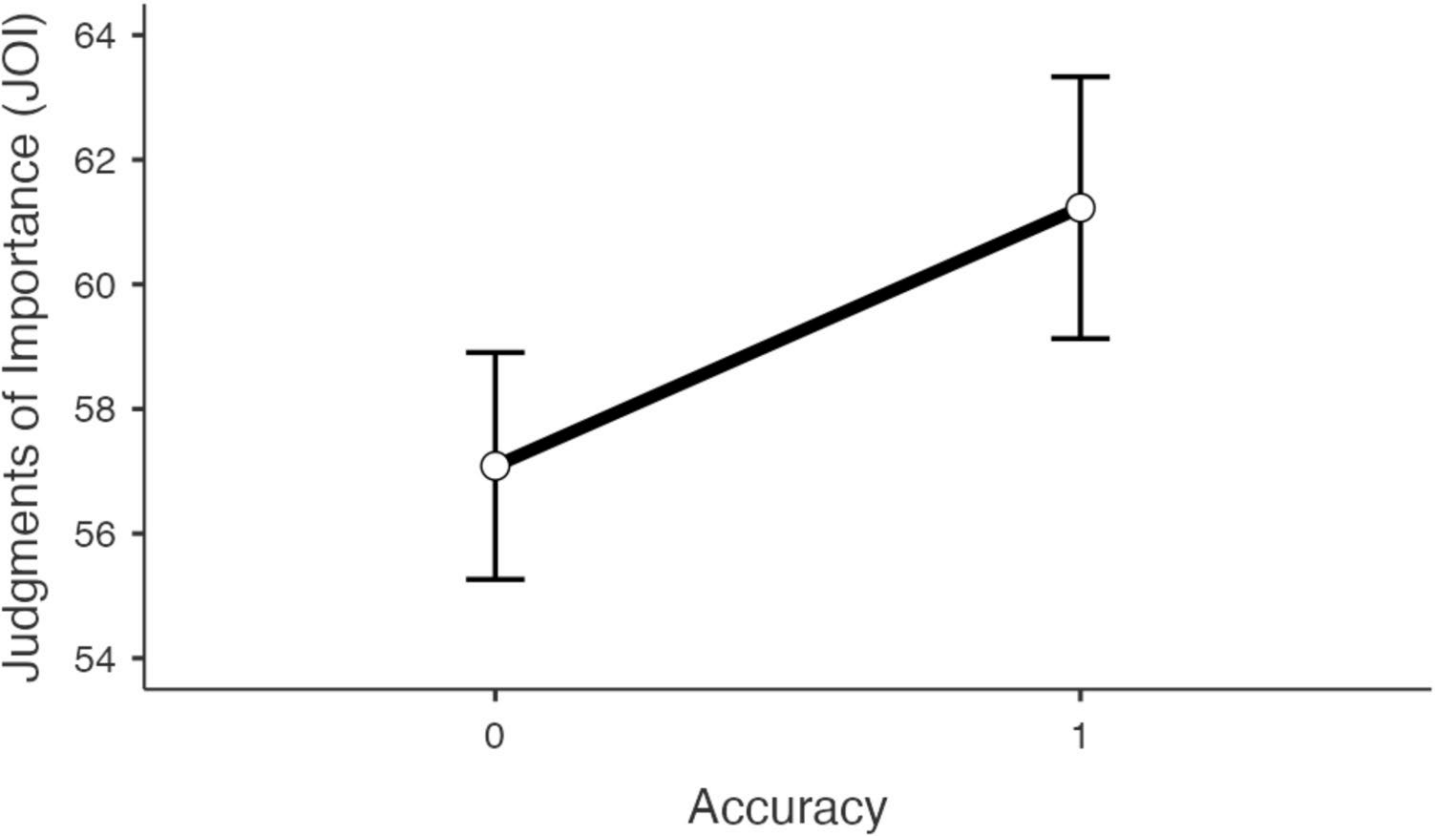
Judgments of importance (JOIs) as a function of recall accuracy while controlling for feeling-of-knowing judgments and each participant’s prior familiarity with each theory in Experiment 3. Error bars reflect the standard error

Judgments of importance as a function of correctness while controlling for feeling-of-knowing judgments and familiarity ratings are shown in Fig. 4. A mixed MLM with item-level JOIs from recall modeled as a function of accuracy, feelings of knowing, and familiarity revealed that accuracy significantly predicted JOIs at recall (*t*(1951) = 2.91, *p* =.004, Estimate: 4.15), such that theories that were correctly recalled were rated as more important (*M* = 68.22, *SD* = 19.36) than theories that were not correctly recalled (*M* = 54.96, *SD* = 18.84). Moreover, FOKs predicted JOIs at recall (*t*(1973) = 9.88, *p* <.001, Estimate:.17), such that the closer a participant felt they were to recalling an item, the more important they rated it. Familiarity also significantly predicted JOIs at recall (*t*(1946) = 4.64, *p* <.001, Estimate:.08), such that theories that participants were more familiar with were rated as more important during recall than theories that participants were less familiar with.

**Fig. 5:**
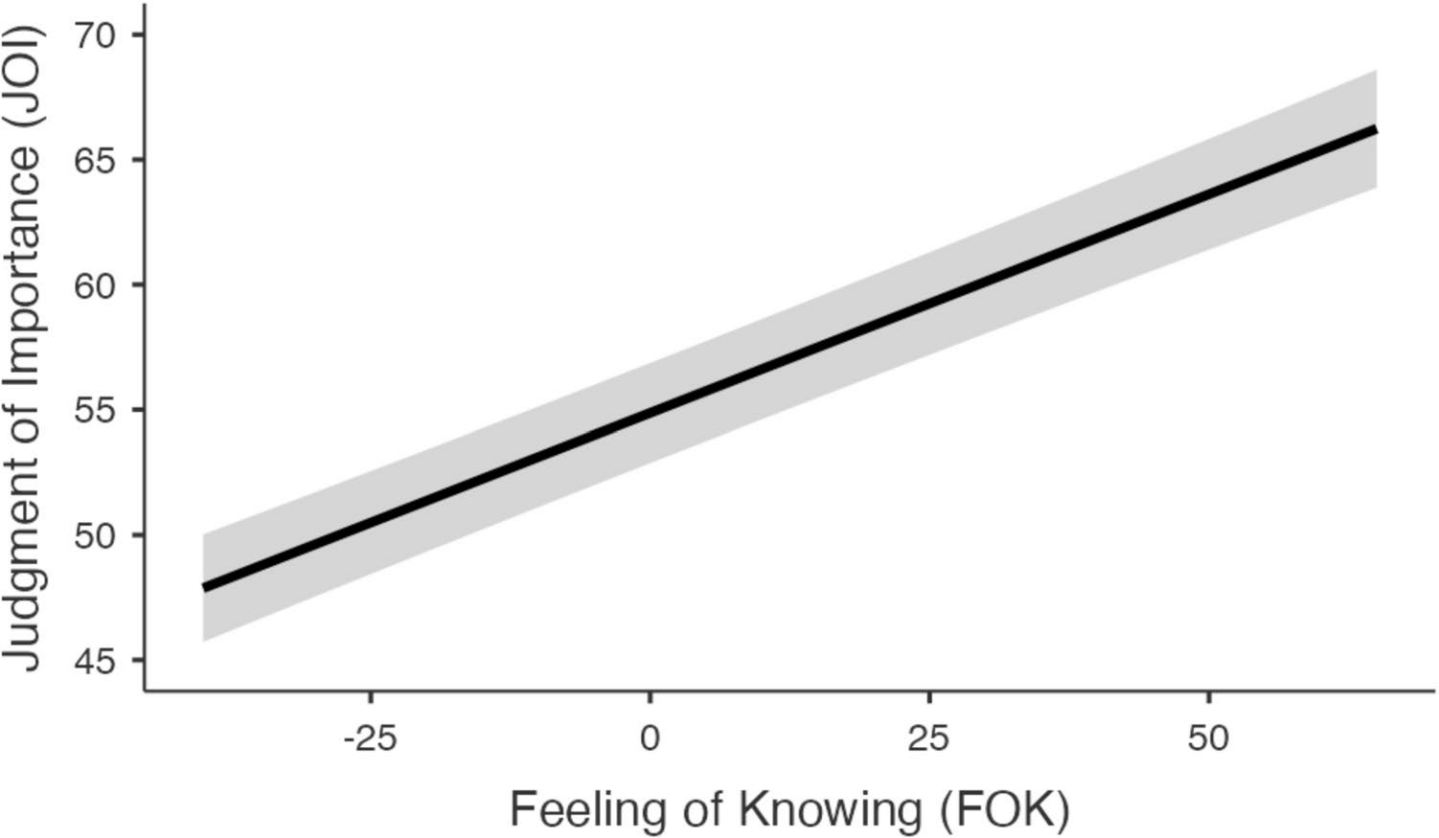
Judgments of importance (JOIs) from incorrect trials as a function feelings-of-knowing judgments while controlling for each participant’s prior familiarity with each theory in Experiment 3. The standard error is shown in gray. We note that due to model centering, the *x*-axis does not necessarily reflect the true scale of the variable

Finally, we also examined only trials where the theory was not correctly recalled (see Fig. 5). A mixed MLM with item-level JOIs from incorrect recall trials modeled as a function of feelings of knowing while controlling for familiarity revealed that feelings of knowing significantly predicted JOIs (*t*(1525) = 8.93, *p* <.001, Estimate:.17), such that the closer a given participant felt they were to recalling a theory, the more important they rated it, even when recall was unsuccessful. Familiarity also significantly predicted JOIs from incorrect recall trials (*t*(1513) = 3.33, *p* <.001, Estimate:.07), such that theories that participants were more familiar with were rated as more important during recall than theories that participants were less familiar with, even on incorrect trials.

### Discussion

In Experiment 3, participants’ feelings-of-knowing judgments were generally accurate, with higher feelings of knowing associated with correctly recalled items compared to items that were not recalled. This accuracy in feelings-of-knowing judgments indicates a reliable metacognitive awareness of memory accessibility. Critically, the results highlighted a significant relationship between feelings-of-knowing judgments and JOIs. Specifically, theories that participants felt were closer to being recalled, as indicated by higher feelings-of-knowing ratings, were judged to be more important, even when actual recall was unsuccessful. This was particularly evident when analyzing incorrect recall trials exclusively, where feelings of knowing still significantly predicted JOIs, demonstrating that the perception of closeness to recall predicts judgments of importance independently of recall success. Thus, Experiment 3 supports the notion of an importance bias predicted by memory accessibility, extending this relationship to situations of retrieval failure.

## Experiment 4

If there is a retrieval-based bias in importance judgments, then reducing the difficulty of the memory test should attenuate this effect. Specifically, it may be that retrieval effort serves as a metacognitive cue for importance. Specifically, when individuals exert more cognitive effort to successfully retrieve information – as in a recall test – the act of retrieval may enhance the perceived importance of that information. In contrast, recognition tests reduce retrieval demands by providing the correct answer as one of several choices, minimizing the sense of effort or accomplishment associated with remembering. If individuals use the subjective experience of retrieval effort or success as a signal of importance, then the importance bias should be weaker or absent in recognition.

As such, in Experiment 4, we employed an experimental manipulation designed to influence the ease of retrieval via differential accessibility. If memory accessibility plays a causal role in judgments of importance, then an experimental manipulation that alters memory accessibility should, in turn, alter judgments of importance as well. Specifically, we compared importance judgments from participants completing a cued recall test with participants completing a recognition test (selecting the correct theory in a four-alternative forced-choice task). We anticipated that the differential memory performance between the recognition test and the cued recall test would lead to variations in judgments of importance as a function of correctness between the two tests. Specifically, given the higher retrieval difficulty/lower memory accessibility in the cued recall test compared to the recognition test, we expected the importance bias for the successfully retrieved items in the cued recall test to be greater than the importance bias for the successfully identified items in the recognition test.

### Method

#### Participants

After exclusions, participants were 202 undergraduate students (*M*_*age*_ = 18.98, *SD*_*age*_ = 1.70 years) – two participants were excluded for cheating and none were excluded for not agreeing to give their full effort.

#### Materials and procedure

The encoding phase in Experiment 4 was identical to Experiment 1, and some participants (*n* = 100) completed a cued recall test with importance judgments at recall only (with no feeling-of-knowing judgments), identical to the procedure in Experiment 1. In contrast, some participants (*n* = 102) were given a recognition test; participants were randomly assigned to conditions. Participants given the recognition test were shown the theory’s definition and three alternative choices (four total answer options) and were asked to choose the correct name for each theory’s definition. All alternative choices were the names of actual theories within the same scientific field, and all of the choices could be potential matches for the given definition (available on the Open Science Framework, see *Data availability*); none of the incorrect alternatives were the names of other studied theories. The order of the questions was randomized for each participant.

### Results

We began by examining potential differences in performance between the two test types. As expected, results revealed that test performance (proportion correct) was much higher for the recognition test (*M* =.74, *SD* =.80) than for the cued recall test (*M* =.25, *SD* =.20), (*t*(200) = −15.76, *p* <.001, *d* = −2.22).

**Fig. 6:**
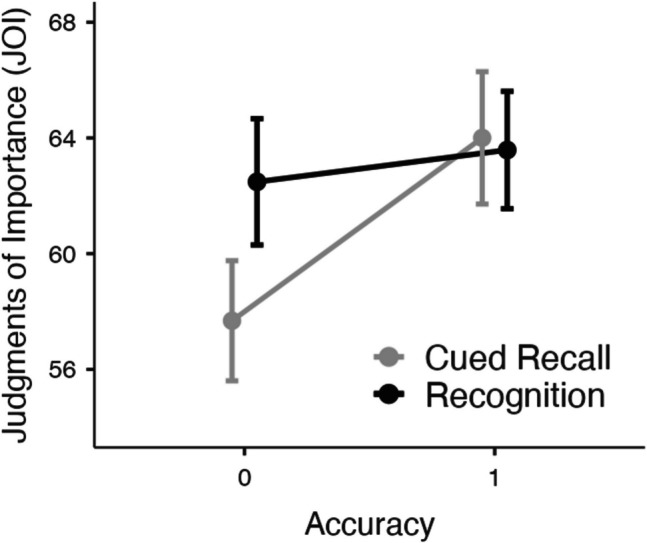
Judgments of importance (JOIs) as a function of accuracy and test type while controlling for each participant’s prior familiarity with each theory in Experiment 4; note that test response time is also in this model. Error bars reflect the standard error

**Fig. 7:**
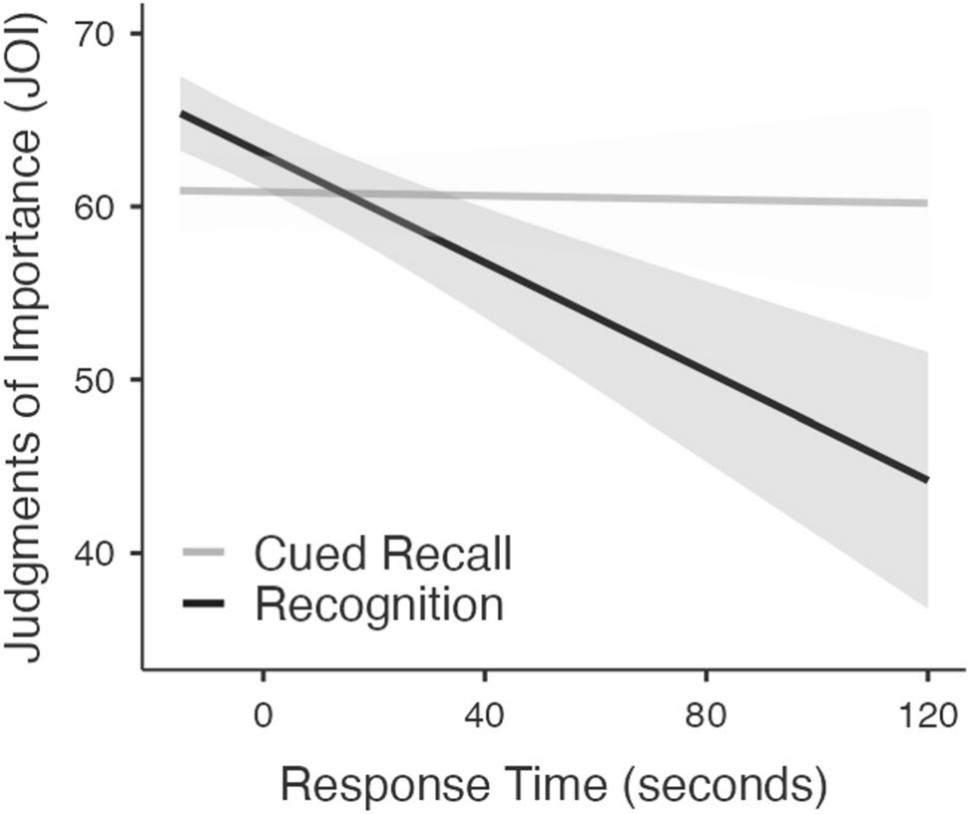
Judgments of importance (JOIs) as a function of response time (seconds) on the test and test type while controlling for each participant’s prior familiarity with each theory in Experiment 4. Standard errors are shown in gray

Next, we examined JOIs for each test type as a function of correctness while controlling for familiarity ratings (see Fig. 6). To better understand whether memory accessibility influences judgments of importance in recognition, per a reviewer’s suggestion, we also analyzed response time on the test (see Fig. 7) as a continuous proxy for retrieval fluency, which may reveal subtle variations in accessibility not captured by binary accuracy alone. To prepare the response time data for analysis, we conducted several preprocessing steps designed to reduce noise from aberrant responses while preserving meaningful individual differences. First, for each participant, we computed *z*-scores for all response times on the memory test to identify within-subject outliers. Any response time with a *z*-score less than –2.5 or greater than 2.5 was recoded as missing, thereby excluding extreme fast or slow responses that likely reflected lapses in attention or other non-task-related influences. This within-subject trimming procedure follows recommendations from modeling work on response time distributions (e.g., Ratcliff, 1993), which emphasize the importance of accounting for individual variability to reduce measurement noise. Next, we aggregated the response time values to compute the mean response time for each participant and identified eight between-subject outliers, which were removed in the response time analysis.

A mixed MLM with item-level JOIs from recall modeled as a function of test type (cued recall, recognition), accuracy, test response time (seconds), and familiarity revealed that JOIs were higher when response times were faster (*t*(3722) = −2.20, *p* =.028, Estimate: -.08). Neither the type of test (*t*(208) =.92, *p* =.358, Estimate: 2.19) nor familiarity predicted JOIs (*t*(3733) = 1.23, *p* =.217, Estimate:.02). JOIs were higher when memory was accurate (*t*(3683) = 4.21, *p* <.001, Estimate: 3.71). Response time did not interact with accuracy (*t*(3612) = -.35, *p* =.727, Estimate: -.02). Test type interacted with accuracy (*t*(3680) = −2.97, *p* =.003, Estimate: −5.22), such that accuracy predicted JOIs during the test when memory was assessed via cued recall (*t*(3675) = 4.88, *p* <.001, Estimate: 6.32) but not when memory was assessed via recognition (*t*(3689) =.92, *p* =.355, Estimate: 1.10). The type of test interacted with response time (*t*(3714) = −2.07, *p* =.039, Estimate: -.15) and an analysis of the simple effects indicated that response time did not predict JOIs on the cued recall test (*t*(3676) = -.12, *p* =.904, Estimate: -.01), but when memory was assessed via recognition, JOIs were greater when response times were faster (*t*(3728) = −2.69, *p* =.007, Estimate: -.16). There was not a three-way interaction between response time, test type, and accuracy (*t*(3609) =.71, *p* =.478, Estimate:.09).

### Discussion

In Experiment 4, we compared two test types, cued recall and recognition, to explore how variations in retrieval difficulty might influence perceived importance. Results revealed that memory accuracy significantly predicted JOIs in the cued recall condition, aligning with the hypothesis that the increased retrieval difficulty yields an importance bias whereby remembered information is considered more important than forgotten information. However, this relationship between memory accuracy and JOIs was not observed in the recognition condition. This suggests that retrieval difficulty may serve as a cue for importance, and memory accessibility during retrieval may drive subjective judgments of importance.

These findings raise an important interpretive question regarding the role of retrieval effort in shaping importance judgments. Effort may signal high importance (e.g., “I worked to retrieve it because it mattered”) or, conversely, low importance (e.g., “If it were important, I would have remembered it easily”). To further explore this issue, we analyzed response time data as a continuous proxy for retrieval fluency. Results revealed that when the answer is present (as in recognition), participants may use their fluency or ease of responding as a cue for importance – quicker responses are perceived as more meaningful. This pattern aligns with the interpretation that fluency, rather than retrieval effort per se, may serve as a weak metacognitive cue for importance, even when retrieval is externally supported. However, it is also possible that recognition is generally perceived as easier, which may reduce the diagnostic value of retrieval experiences for informing judgments. Future work may benefit from directly manipulating perceived effort or diagnosticity to clarify whether effort enhances or undermines importance judgments.

## General discussion

In the present study, we examined potential biases in subjective assessments of importance based on memory outcomes. Specifically, participants were tasked with learning 20 scientific theories for a later test. Following the study phase, participants were asked to recall the names of the theories based on their definitions. Immediately after each recall attempt, participants provided a judgment of importance (JOI) for each theory. Participants also rated their prior familiarity with each theory after the memory test to control for pre-existing knowledge. The present experiments encompassed various experimental conditions, including different memory test formats (cued recall and recognition) and the inclusion of metacognitive judgments (feelings of knowing) to examine their influence on the relationship between memory retrieval outcomes and perceptions of importance. By examining the relationship between memory outcomes and subjective assessments of importance, we sought to uncover whether retrieval success predicts how individuals perceive the importance of information.

Results revealed a retrieval-based bias in participants’ evaluations of importance. Specifically, theories that were successfully recalled were rated as more important than those not recalled, even when controlling for prior familiarity with a theory (which also affected importance ratings such that theories that participants were more familiar with (and thus more accessible in memory) were also rated as more important). Additionally, even when participants failed to recall a theory, the closer they were to retrieval (as indicated by feelings of knowing), the more important they rated that theory. By exploring the relationship between metacognitive feelings of knowing and importance judgments, we gained deeper insights into the mechanisms underlying the observed importance bias and its connection to memory processes during retrieval. Namely, the more accessible an item is in memory, the more important it is considered to be.

To further validate these effects, we used multilevel models that included random intercepts for both participants and items. This analytic approach allowed us to account for variability not only across individuals but also across the specific scientific theories used as stimuli (Judd et al., 2012). By including item-level random intercepts, we ensured that the observed effects were not driven by idiosyncratic features of particular theories (e.g., some being more inherently memorable or familiar than others). This strengthens the generalizability of our findings and supports the interpretation that the importance bias reflects cognitive mechanisms tied to retrieval and accessibility, rather than stimulus-specific confounds.

To test whether the importance bias is influenced by retrieval difficulty, we employed two different testing conditions: half of the participants performed a recall test while the other half engaged in a recognition test. Not surprisingly, accuracy for recognition exceeded accuracy for recall which is at least partially due to differences in accessibility between the two test formats (Tulving & Pearlstone, 1966). Critically, when memory was tested via recognition (where the retrieval process is different based on being given an exact cue rather than having to produce the cue on your own), there were no differences in JOIs as a function of accuracy. Thus, when the to-be-retrieved information is accessible (i.e., the correct information was present on the screen), learners do not impart this importance bias. Rather, there must be an attempt to consciously recollect (whether successful or not) for the learner to use the accessibility of information in memory as a proxy for its importance. Although accuracy did not predict JOIs in the recognition condition – likely because the correct answer was visible on the screen – faster response times were associated with higher JOIs. This suggests that both overt recollection (e.g., recall) and more implicit forms of accessibility (as indexed by response fluency) can shape importance judgments.

Our demonstration of this *importance bias,* where remembered information is judged as more important than forgotten information, could be underpinned by several cognitive and metacognitive mechanisms. On a fundamental level, this bias may stem from our inherent drive for cognitive efficiency – we naturally prioritize the encoding, storage, and retrieval of information deemed crucial for survival and goal attainment (for adaptive memory views, see Nairne & Pandeirada, 2008, 2010; Nairne et al., 2017, 2019). For example, *need probability* pertains to the expected future necessity or examination of specific information (Anderson & Schooler, 1991, 2000; Anderson et al., 1997). It is theorized that in practical situations, this need probability tends to wane as time progresses, resulting in a parallel decline in memory accessibility for the information in question. This pattern of forgetting is thought to align with the decreasing likelihood of needing the information. Cognitive efficiency is thus achieved by enhancing the accessibility of likely-to-be-needed important information, leading to a correlation between memory performance and perceived importance (see Murphy & Castel, 2020, 2021a, b).

Considering remembered information as more important than forgotten information may also stem from processes similar to those described by the availability heuristic (Schwarz et al., 1991; Tversky & Kahneman, 1973). While the availability heuristic traditionally explains how easily recalled information can bias judgments of frequency or likelihood, similar accessibility-based mechanisms may influence perceived importance. That is, if something is easily remembered, people may not only judge it as more frequent, but also infer that it is more meaningful. This heuristic can skew our judgment and decision-making processes, as demonstrated by participants in Experiment 3 rating theories as more important when also providing higher feelings of knowing (a proxy of memory accessibility) for those theories. The present findings also align with prior work showing that successful retrieval can influence downstream evaluative judgments, such as Grybinas and Dobbins (2021), who found that retrieving associative information like names and professions led to higher attractiveness ratings for faces compared to those for which recall failed (see also Grybinas et al., 2019). This suggests a broader pattern in which the accessibility of information during retrieval biases not only perceptions of importance but also evaluations of unrelated attributes.

From a metacognitive perspective, individuals might interpret their ability to remember information as a direct indication of its importance. This interpretation could be influenced by the feeling-of-knowing judgments where even if the information is not immediately accessible, the underlying sense of familiarity or closeness to retrieval could enhance the perceived importance of the information. This suggests that learners might be using memory accessibility as a heuristic for judging importance, reinforcing the link between retrieval success and perceived value. Additionally, successful retrieval may be intrinsically rewarding, as it signals both the completion of a valued goal and a demonstration of one’s cognitive proficiency. This rewarding experience may, in turn, amplify the perceived importance of the retrieved information, reinforcing the link between memory success and subjective value. This feedback loop could strengthen the association between memory accessibility and perceived importance over time. As individuals repeatedly experience retrieval success with certain pieces of information and subsequently judge them as important, these judgments could further enhance the accessibility of this information in memory, creating a self-reinforcing cycle.

The present findings are best understood through the lens of metacognitive inferences rooted in accessible lay theories of cognition (Schwarz, 2010, 2015). As Schwarz and colleagues have shown, people frequently draw judgments from the subjective dynamics of their cognitive experiences, such as the ease or difficulty of retrieval, by applying implicit theories about how memory functions. Our results align with this framework: participants likely used memory accessibility as a cue to infer importance based on the common lay theory that important things are easier to remember. Importantly, this inference is more consistent with judgments of personal or subjective importance rather than objective or domain-general significance. That is, the theories participants judged as important may have simply felt relevant or familiar based on their accessibility, not because of their actual value within the scientific canon. This interpretation helps explain why our effects were observed even when controlling for familiarity, and why fluency-based judgments (e.g., feelings of knowing) also predicted importance. Together, our data support the view that judgments of importance can be shaped by metacognitive experiences and may reflect inferences from accessible – but not necessarily diagnostic – cues, consistent with past work on ease-of-retrieval manipulations (Haddock et al., 1999; Winkielman & Schwarz, 2001) and observer-based inferences (Smith & Schwarz, 2016).

The present findings suggest that both retrieval success and metacognitive experiences of accessibility, such as feelings of knowing, contribute to judgments of importance, and these may reflect a common underlying mechanism. Specifically, our data align with the view that subjective experiences of accessibility, rather than accuracy per se, inform evaluations of informational value. Successful retrieval likely co-occurs with a sense of fluency or ease, which may serve as a cue for importance. Similarly, even when recall fails, elevated feeling-of-knowing judgments predict higher importance ratings, indicating that accessibility-based experiences – whether tied to actual recall or the felt potential for retrieval – shape perceived importance. This interpretation is consistent with Haddock et al. (1999), who argue that metacognitive experiences, including feelings of familiarity and accessibility, influence evaluative judgments. Thus, across our experiments, we interpret retrieval success, feeling-of-knowing judgments, and related fluency experiences as converging cues that reflect – and reinforce – the broader metacognitive heuristic that accessible information feels more important.

While our findings consistently suggest that accessibility influences judgments of importance, the results of Experiment 2 complicate this narrative. When importance ratings were collected at both encoding and retrieval, initial JOIs predicted later ones, and the effect of retrieval success on recall-phase JOIs was no longer significant. This suggests that memory accessibility during retrieval may reinforce or validate earlier beliefs about importance, rather than fully override them. In contrast, when JOIs were collected only at retrieval (Experiments 1, 3, and 4), retrieval success consistently predicted perceived importance. Together, these findings point to a more dynamic relationship between encoding- and retrieval-based processes in shaping importance judgments, such that subjective accessibility may interact with, rather than solely determine, evaluations of value.

An alternative interpretation of our findings is that judgments of importance reflect a value signal that influences memory encoding such that more important information is encoded more effectively and subsequently recalled more successfully. From this perspective, successful retrieval would correlate with higher importance judgments not because of a retrieval-based inference, but because importance (as a proxy for value) facilitated stronger encoding. While our results showed that memory accessibility – particularly during recall – is associated with elevated JOIs, we acknowledge that encoding- and retrieval-based processes are deeply intertwined and may co-influence both memory performance and metacognitive judgments. This conceptual entanglement reflects a longstanding theoretical challenge in distinguishing the functional contributions of value, encoding, and retrieval. Our finding that encoding-phase JOIs did not predict later recall accuracy, and that differences between encoding and retrieval JOIs were not modulated by retrieval success, weighs against a purely encoding-driven account. However, the response time data from Experiment 3 – showing that faster recognition responses were associated with higher JOIs – raises the possibility that fluency-based signals, possibly originating from encoding strength, may shape importance judgments even when overt recall is not required. Thus, while we emphasize retrieval-based metacognitive inferences as a primary mechanism, our data remain consistent with a complementary view in which perceived importance facilitates encoding and later retrieval success, suggesting a bidirectional relationship between value and memory processes.

One important limitation of the present work concerns how participants may have interpreted the term “importance” when making their judgments. Although our question asked how important each theory was “for our understanding of the world,” this phrasing may have been interpreted as reflecting personal importance rather than objective or domain-specific importance. That is, participants may have based their judgments on how relevant, meaningful, or memorable the theory was to them personally, rather than on its broader scientific or societal significance. This is important because the metacognitive implications differ: personal importance is more likely to influence and be influenced by memory processes, whereas judgments of general importance (e.g., in fields outside one’s expertise) may not be as tightly linked to accessibility. Thus, our findings are most appropriately interpreted as reflecting subjective or personal importance, which can be biased by memory accessibility. Future research could more explicitly distinguish between personal and general importance to examine whether accessibility-based biases persist across different conceptualizations of value.

Another limitation of the present study concerns the timing of when participants form their judgments about the importance of the scientific theories. Although participants made importance judgments during the test phase (or after encoding each theory in Experiment 2), there is a possibility that participants may have engaged in some form of selective encoding for the theories they considered important, rather than JOIs being influenced by memory accessibility during the test. However, participants were told to study each theory as if they were preparing for a typical course examination (i.e., remember as much as you can). Thus, participants did not have any incentive to try and better remember important theories. Additionally, there was no evidence that importance ratings during encoding influenced recall. However, future work could include an incidental encoding condition that provides JOIs as they view each theory without mention of any memory test. Comparing JOIs during recall from this group to those who expect a test could help rule out selective encoding as a potential explanation for the observed importance bias.

Regardless of whether the importance bias originates at encoding, retrieval, or through a combination of both, what is clear from the current study is that it consistently *manifests at recall*. This has important real-world implications: when individuals evaluate the importance of information – such as when reviewing grant proposals, recalling key points in a meeting, or making decisions based on past events – the accessibility of that information in memory can subtly bias their judgments. Rather than being an objective assessment of value, these judgments may reflect the perceived importance *as inferred from memory success*. In this way, retrieval becomes a critical moment where subjective importance is shaped, potentially reinforcing a cycle where remembered information is judged as more important simply because it comes to mind more easily.

The present findings have implications for how we reconstruct past and simulate future experiences. People are constantly re-evaluating their judgments of importance for their previous experiences and these judgments seem largely dependent on memory accessibility for those experiences. This might have direct implications within the domain of science. As noted earlier, the US Department of Health and Human Services (2013) reports different types of biases involved in evaluating the importance of proposed research, like the country of origin of the research or the institute where the research was conducted. Our results suggest an additional bias involved in evaluating the importance of the proposed research, which is how memorable the proposal is relative to the other proposals. For instance, imagine submitting a grant for review. Also, imagine having the option to write it in a bullet point format or a narrative format. Prior research has demonstrated that narrative increases memorability (Yarkoni et al., 2008). According to our results, the grant written in a narrative format will be remembered better and as a result, be rated as more important than the grant written in bullet point format. Therefore, the memorability of your grant might determine whether you eventually received that grant. Our findings might also have implications for the way people construct priority lists and the biases involved in that process (i.e., a metacognitive control process implementation derived from the metacognitive monitoring process of a judgment of importance).

In sum, the present experiments were designed to investigate the presence of a retrieval-based bias in judgments of importance. Our central hypothesis was that JOIs for the theories whose names were successfully retrieved would be significantly higher than the JOIs for the theories whose names were not successfully retrieved. As predicted, when participants could access their memory for the name of a theory, their judgments about how important that theory was for our understanding of the world increased. Thus, people develop a bias regarding the importance of remembered versus forgotten information, potentially leading them to judge remembered information as more important than forgotten information. While previous work has shown that people regard memory as a process sensitive to the importance of information (see Murphy, 2025), the present study suggests that people may hold an erroneous belief that the reverse case is also true. That is, people may deem things they remember as having higher importance and things they forget as having less importance, based in part on the degree of memory accessibility which is not necessarily a valid indicator of the true status of that information’s value.

## Data Availability

The data and materials used in this study are available at the Open Science Framework (OSF): https://osf.io/huv2f/?view_only=1e7f422f5f244f3a979f822ab79116f6
